# The complex interplay between clinical and person-centered diabetes outcomes in the two genders

**DOI:** 10.1186/s12955-017-0613-0

**Published:** 2017-02-21

**Authors:** Maria Chiara Rossi, Giuseppe Lucisano, Basilio Pintaudi, Angela Bulotta, Sandro Gentile, Marco Scardapane, Soren Eik Skovlund, Giacomo Vespasiani, Antonio Nicolucci, on behalf of the BENCH-D Study Group, A. Nicolucci, G. Vespasiani, M. C. Rossi, S. Gentile, A. Bulotta, S. E. Skovlund, E. Forte, F. Tuccinardi, A. Griffo, S. Leotta, L. Fontana, M. Altomare, L. Pellegrini, F. Malci, C. Moscatelli, P. Tatti, M. Neri, G. Santantonio, F. Chiaramonte, R. A. Rabini, S. Rosati, F. D’Angelo, G. Maolo, B. Polenta, S. Lardelli, A. M. Tesi, L. Cotti, G. Garrapa, R. Viola, M. Manuela, F. Lizzadro, M. G. Cartechini, N. Busciantella Ricci, G. Agostinelli, I. Meloncelli, M. Galetta, V. Marconi, L. Carini, I. Crema, L. Clementi, S. Manfrini, L. Olivi, P. Foglini, R. Maricotti, P. Pantanetti, A. Spalluto, M. Andreani, G. Martinelli, A. Chiambretti, R. Fornengo, L. Di Vito, M. Albertone, V. Magliano, D. Cortale, A. R. Bogazzi, M. Rivelli, S. B. Del Rosso, F. Picataggi, P. Bonfani, E. Baccaro, M. Comoglio, R. Manti, O. Boscolo, C. Laiolo, A. Clerico, L. Richiardi, K. Sinato, G. P. Carlesi, S. Garrone, G. Magro, C. Paverin, D. Gaviglio, G. Saglietti, L. Monge, G. Grassi, A. Di Benedetto, M. Russo, B. Pintaudi, G. Di Vieste, A. Garofalo, F. Vitale, L. Bernardo, G. Saitta, A. Lo Presti, M. A. Fulantelli, G. Mattina, M. Cortese, A. Parrinello, V. Provenzano, L. Ferrara, R. Ferranti, D. Gioia, M. Conti, G. Lucisano, M. Scardapane, M. Valentini, D. D’Alonzo, C. Pirozzoli, R. Memmo, B. Di Nardo, R. Chiodo, C. De Francesco

**Affiliations:** 1 CORESEARCH - Center for Outcomes Research and Clinical Epidemiology, Pescara, Italy; 2 S.S.D. Diabetologia, Niguarda Ca’ Granda Hospital, Milan, Italy; 3 Novo Nordisk SpA, Rome, Italy; 4 Department of Clinical and Experimental Medicine, Second University of Naples, Naples, Italy; 5 Novo Nordisk A/S, Soeborg, Denmark; 6 Diabetes Unit, Madonna del Soccorso Hospital, San Benedetto del Tronto, AP Italy

**Keywords:** Type 2 diabetes, Gender-disparities, Diabetes-related distress, Psychological wellbeing

## Abstract

**Background:**

New approaches to cope with clinical and psychosocial aspects of type 2 diabetes (T2DM) are needed; gender influences the complex interplay between clinical and non-clinical factors. We used data from the BENCH-D study to assess gender-differences in terms of clinical and person-centered measures in T2DM.

**Methods:**

Clinical quality of care indicators relative to control of HbA1c, lipid profile, blood pressure, and BMI were derived from electronic medical records. Ten self-administered validated questionnaires (SF-12 Health Survey; WHO-5 well-being index; Problem Areas in Diabetes (PAID) 5, Health Care Climate Questionnaire, Patients Assessment of Chronic Illness Care, Diabetes Empowerment Scale, Diabetes Self-care Activities, Global Satisfaction for Diabetes Treatment, Barriers to Taking Medications, Perceived Social Support) were adopted as person-centered outcomes indicators.

**Results:**

Overall, 26 diabetes clinics enrolled 2,335 people (men: 59.7%; women: 40.3%). Lower percentages of women reached HbA1c levels < =7.0% (23.2% vs. 27.8%; *p* = 0.03), LDL-cholesterol < 100 mg/dl (48.3 vs. 57.8%; *p* = 0.0005), and BMI <27 Kg/m2 (27.2 vs. 31.6%; *p* = 0.04) than men. Women had statistically significant poorer scores for physical functioning, psychological well-being, self-care activities dedicated to physical activities, empowerment, diabetes-related distress, satisfaction with treatment, barriers to medication taking, satisfaction with access to chronic care and healthcare communication, and perceived social support than men; 24.8% of women and 8.8% of men had WHO-5 < =28 (likely depression) (*p* < 0.0001); 67.7% of women and 55.1% of men had PAID-5 > 40 (high levels of diabetes-related distress) (*p* < 0.0001). At multivariate analysis, factors associated with an increased likelihood of having elevated HbA1c levels (≥8.0%) were different in men and women, e.g. having PAID-5 levels >40 was associated with a higher likelihood of HbA1c ≥8.0% in women (OR = 1.15; 95%CI 1.05–1.25) but not in men (OR = 1.00; 95%CI 0.93–1.08).

**Conclusions:**

In T2DM, women show poorer clinical and person-centered outcomes indicators than men. Diabetes-related distress plays a role as a correlate of metabolic control in women but not in men. The study provides new information about the interplay between clinical and person-centered indicators in men and women which may guide further improvements in diabetes education and support programs.

## Background

The considerable pressure on health care systems to provide high-quality care while controlling costs has led several public and private health care organizations to promote initiatives to measure and improve the quality of care for people with diabetes [1, 2].

In Italy, the Associazione Medici Diabetologi (AMD) scientific society has implemented since 2006 a continuous improvement effort involving a large network of diabetes clinics throughout the country (AMD-Annals) [3]. The periodic dissemination of Annals has been effective in improving several process and intermediate outcome indicators clinical indicators over a few years [4]. The model has also been proven to be cost-effective [5].

Data from AMD-Annals have been recently used to evaluate gender differences in pharmacological and non-pharmacological treatment of diabetes [6]. The analysis was important to demonstrate that gender disparities are less pronounced in Italy than in other countries, but that the likelihood to reach specific clinical outcomes is systematically unfavorable for women as compared to men; in particular, women were 14% more likely than men to have HbA1c levels >9.0% in spite of insulin treatment, 42% more likely to have LDL-cholesterol ≥130 mg/dl in spite of lipid-lowering treatment, and 50% more likely to have BMI ≥30 Kg/m2. These findings suggested that a complex interplay among biological, clinical and behavioral differences can underlie these differences and call for diversifying the care and specializing the support provided to men and women.

In parallel, the increasing recognition of patient-centered care as the best model to ensure a care respectful of, and responsive to patient preferences, needs, and values [7] call for the need to include psychosocial aspects in the quality model of diabetes care, as clearly emphasized by the international Diabetes Attitudes, Wishes, and Needs (DAWN-2) Program [8]. The DAWN-2 study well documented that people with diabetes have major psychosocial issues; in particular, 44.6% (country range 17.2–67.6%) of people with diabetes have diabetes-related distress (i.e. Problem Areas in Diabetes Scale 5 (PAID-5) score ≥ 40) [9], while 13.8% have likely depression [WHO-5 Well-Being Index (WHO-5) score ≤ 28] [10] (country range 6.5–24.1%). Furthermore, diabetes had a negative impact on physical health and social relationship; approximately 40% of participants reported that their medication interfered with their ability to live a normal life.

Due to this body of evidence, individualized interventions based on patient needs, concerns, and capabilities have been promoted while taking gender into account.

In the context of the AMD-Annals and the DAWN2 initiatives, the BENCH-D study (Benchmarking Network for Clinical and Humanistic Outcomes in Diabetes) was launched aiming to test a model of regional benchmarking to monitor and improve not only clinical indicators, but also person-centered outcomes [11]. Besides the AMD clinical indicators for the evaluation of quality of care, the BENCH-D study used validated questionnaires, largely derived from the DAWN2 study, for measuring person-centered dimensions.

The first important step of the BENCH- study was to describe the person-centered indicators in type 2 population and to explore the relationship among different quality of care and quality of life dimensions. As described in two previous papers [12, 13], BENCH-D documented that: high levels of diabetes distress are common among people with type 2 diabetes (T2DM), affecting almost two-thirds of patients; high diabetes distress is associated with worse clinical and psychosocial outcomes; higher empowerment is on the other hand associated with better glycemic control, psychosocial functioning and perceived access to person-centered chronic illness care.

In the present secondary analysis of the BENCH-D data, database has been used to assess gender-differences in T2DM in terms of diabetes-related distress, physical and psychological well-being, empowerment, perceived social support and other measures of satisfaction with treatment and care. We wanted to test the hypothesis that systematic differences exist in the two genders in the interplay between clinical and person-centered indicators, especially on the likelihood on poor metabolic control (i.e. HbA1c > =8.0%). A deeper comprehension of these differences may inform individualized, gender-specific educational approaches.

## Methods

A detailed description of the BENCH-D study protocol and of the questionnaires utilized as person-centered indicators was published elsewhere [11]. Briefly, a random sample of patients with T2DM stratified by diabetes treatment (oral agents, insulin + oral agents, insulin) was selected by 26 diabetes outpatient clinics in Italy.

Following the AMD-Annals methodology [3–6], clinical data were extracted from electronic databases of diabetes clinics, including information on body mass index (BMI), diabetes duration, HbA1c, blood pressure and lipid profile values, glucose-lowering, antihypertensive and lipid-lowering treatments, diabetic complications (i.e. retinopathy, diabetic nephropathy, foot complications, and previous cardiovascular and cerebrovascular events). AMD-Annals intermediate outcome measures were evaluated on the BENCH-D sample; these indicators include the proportion of patients with satisfactory values as well as the percentage of those with unacceptably high values. Outcomes were considered satisfactory if HbA1c levels were ≤7.0% (≤53 mmol/mol), blood pressure values were ≤130/80 mmHg, LDL cholesterol (LDL-c) levels were <100 mg/dl, and BMI was <27 Kg/m^2^. Unsatisfactory outcomes include HbA1c levels >8.0%, blood pressure values ≥140/90 mmHg, LDL levels ≥130 mg/dl, and BMI ≥30 Kg/m^2^ [6].

Information on socio-demographic characteristics, quality of life, satisfaction, and self-care behaviors and attitudes was collected using an ad hoc self-administered questionnaire including ten validated instruments which are described in Table 1 [9, 10, 14–32]. The scores of these instruments represented the person-centered outcomes. In line with the methodology applied in the DAWN2 study [8], the instruments were chosen to evaluate the impact of diabetes and its management on physical and psychological well-being and satisfaction. In addition, perceived barriers, diabetes distress, and social support were included as mediators of the relationship between self-care activities, quality of life, and diabetes outcomes. All the instruments were validated and showed satisfactory psychometric properties [9].

**Table 1:** Questionnaires used and validated in the BENCH-D study

Questionnaire	Abbreviation	Domain	Brief description	No. of items	Scoring	References
SF-12 Health Survey - physical component	SF-12 PCS	Physical functioning	SF-12 is a widely used generic health status measure. It includes 12 items which can be aggregated into two summary measures: the Physical (PCS) and Mental (MCS) Component Summary scores. Both scores range from 0 (worst possible health state) to 100 (best possible health state); they are normalized to a general population mean of 50 and an SD of 10.	6	0-100	[14]
WHO-5 well-being index	WHO-5	Psychological well-being	WHO-5 assesses the psychological well-being, a core component of overall quality of life. It is also a valid and reliable risk assessment measure for mild, moderate and severe depression. A score <50 indicates poor psychological well-being, a score <=28 indicates probable depression.	5	0-100	[10, 15]
Problem Areas in Diabetes	PAID-5	Diabetes distress	PAID-5 evaluates diabetes related emotional distress, i.e. specific worries and negative emotions related to diabetes. A score >40 indicates high diabetes-related distress.	5	0-100	[9, 16–20]
Health Care Climate Questionnaire - Short Form	HCC-SF	Person centered communication	HCC-SF evaluates the extent to which clinicians tend to favor the autonomy of the patient or, instead, tend to assume a paternalistic attitude towards the patient. Higher scores correspond to a higher perception by the patient of autonomy support.	6	0-100	[21, 22]
Patients Assessment of Chronic Illness Care - Short Form	PACIC-SF	Quality of chronic illness care and patient support	PACIC provides an assessment of patient perceived access to support from the health care team according to a chronic care health delivery model. The higher the score the more favorable the patient experience.	11	0-100	[23, 24]
Diabetes Empowerment Scale - Short Form	DES-SF	Diabetes Psychosocial Self-Efficacy	DES-SF assesses the patient’s confidence in taking an active role in own management of the condition. The higher the score the higher the patient empowerment	8	0-100	[25, 26]
Diabetes Self-care Activities	SDSCA-6	Self-care activities	SDSCA-6 assesses self-reported health behaviors related to diet, physical activity, self-monitoring of blood glucose, foot care and medication taking. Each item is reported individually.	6	0-7	[27]
Global Satisfaction for Diabetes Treatment	GSDT	Satisfaction with treatment regimen	GSDT assesses overall satisfaction with the medical diabetes treatment, here under the perceived impact of medication on daily life and psychological well being, The higher the score the higher the treatment satisfaction .	7	0-100	[28]
Barriers to Taking Medications	BM	Barriers to taking medication	BM assesses what concrete barriers patients feel they face in daily life to taking their medication as scheduled. The higher the score the higher the perceived barriers.	10	0-100	[29–32]
Perceived social support	PSS	Patient perceived support	PSS assesses satisfaction with social support from various sources for managing diabetes (healthcare system, community, family, peers…). The higher the score the higher the perceived support.	5	0-100	[29–32]

All the scores (i.e. person-centered indicators) ranged between 0 and 100, with higher values indicating higher levels of the dimension investigated. The only exception is the SDSCA6 scale [27] that provides a single score for each item, ranging from 0 to 7 to indicate the average number of days in the previous week respondent had performed each self-care activity. Two dichotomous person-centered indicators were also utilized: percentage of people with WHO-5 < =28 indicating likely depression [10, 15], and percentage of people with PAID-5 > 40 indicating high diabetes-related distress [9, 16–20].

All the clinical and person-centered data collected in the study have been anonymized and centrally analyzed. Local ethics committees of all participating centers approved the protocol.

### Statistical analysis

No formal sample size estimation was performed. Each center was required to enroll up to 100 patients.

Differences in socio-demographic characteristics, clinical outcomes and in mean quality of life/satisfaction scores according to gender were evaluated and compared using the chi-square tests for categorical variables and the Mann-Whitney test for continuous variables. Data were expressed as mean and standard deviation or frequency. A p-value <0.05 was considered statistically significant.

Mean scores of questionnaires were also adjusted for age, diabetes duration, BMI, presence of diabetes complications, glucose-lowering treatment class, school education, and living status. Data were expressed as mean and standard error.

Fully adjusted multivariate logistic models, separated for men and women, were applied to identify socio-demographic, clinical and person-centered factors associated with the achievement of HbA1c levels ≥8.0% in the two genders. Results are expressed as Odds Ratios (ORs) with their 95% confidence intervals (95%CI). All statistical analyses were performed using SAS 9.4 (The SAS Institute, Cary, NC, USA),

## Results

**Fig. 1:**
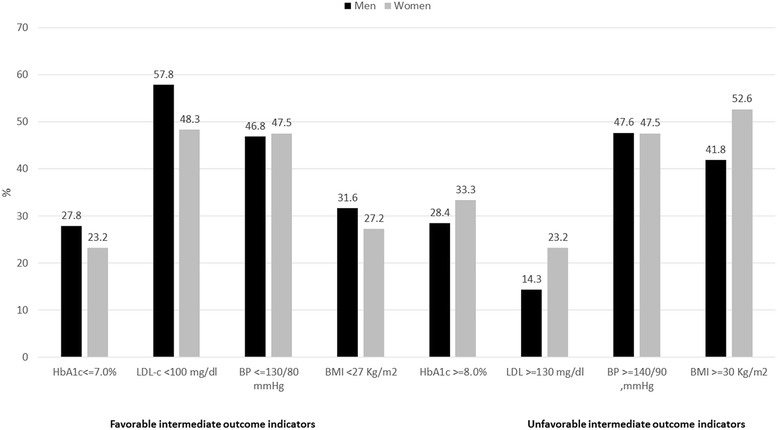
Between-gender differences in the intermediate outcomes indicators of AMD-Annals

Between January 2010 and July 2011, 26 diabetes clinics enrolled 2,390 people with T2DM; information on gender was available for 2,335 (97.7%); men were 1,393 (59.7%) and women were 942 (40.3%). Population characteristics by gender are reported in Table 2: compared to men, women were slightly older, had a lower level of school education, and were more likely to live alone. Women were less frequently smokers than men. From a clinical point of view, despite a longer diabetes duration and a higher likelihood to be treated with insulin, women showed a lower prevalence of known diabetes complications than men. Higher levels of HbA1c, total and LDL-cholesterol and BMI were found in women and AMD-Annals intermediate outcome indicators were systematically less satisfactory in women than in men, with lower percentages of women reaching favorable therapeutic goals for HbA1c, lipid profile and BMI than men, and parallel higher percentages of women with unacceptably high levels of the same parameters (Fig. 1).

**Table 2:** Socio-demographic and clinical characteristics according to gender

		Men	Women	p ^a^
N		1393	942	
%		59.7	40.3	
Socio-demographic characteristics				
Mean age (years)		64.4 (10.0)	66.1 (10.4)	<0.0001
Age in classes (%)	<55	17.5	14.8	<0.0001
	55–65	33.3	27.6	
	65–75	35.3	37.8	
	> = 75	13.9	19.7	
School education (%)	Primary school	30.3	53.4	<0.0001
	Middle school	32.7	22.9	
	High school	28.4	19.8	
	University	8.6	3.9	
Working status (%)	Employed	30.5	12.0	0.77
	Housewife	0.7	34.8	
	Retired	65.8	51.9	
	Unemployed/student	3.1	1.3	
Living status (%)	Alone	8.7	16.7	<0.0001
	Spouse/sons	84.8	79.8	
	Other	6.5	3.4	
Clinical characteristics				
Smokers	No	33.5	73.2	<0.0001
	Yes	20.2	11.4	
	Ex	46.3	15.5	
BMI (Kg/m^2^)		29.7 (5.9)	31.0 (6.1)	<0.0001
Diabetes duration (years)		13.4 (15.1)	15.5 (15.6)	0.006
Mean HbA1c (%)		7.6 (1.5)	7.8 (1.5)	0.002
Total cholesterol (mg/dl)		170.9 (38.1)	187.3 (40.4)	<0.0001
LDL cholesterol (mg/dl)		96.9 (31.1)	106.7 (34.4)	<0.0001
HDL cholesterol (mg/dl)		46.7 (13.9)	54.1 (14.2)	<0.0001
Triglycerides (mg/dl)		142.8 (116.4)	140.0 (100.6)	0.64
Systolic blood pressure (mmHg)		135.1 (15.8)	135.8 (16.8)	0.39
Diastolic blood pressure (mmHg)		78.3 (9.3)	78.2 (8.8)	0.97
Diabetes treatment (%)	Oral agents	52.6	44.9	0.001
	Oral agents + insulin	23.0	27.2	
	Insulin	24.3	27.9	
Lipid-lowering treatment (%)		48.5	47.6	0.65
Antihypertensive treatment (%)		67.6	70.4	0.16
No. of diabetes complications	0	63.7	70.0	0.0001
	1	26.1	23.9	
	> = 2	10.2	6.2	

^a^ χ-square test for categorical variables and the Kruskal-Wallis test for continuous variables

**Fig. 2:**
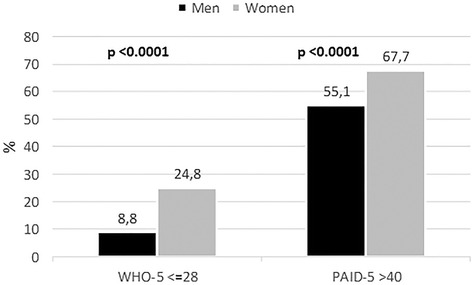
Between-gender differences in the prevalence of likely depression (WHO-5 < =28) and high diabetes-related distress (PAID-5 > 40)

Between-gender differences in person-centered indicators are shown in Table 3. Women had statistically significant poorer scores for physical functioning, psychological well-being, self-care activities dedicated to physical activities, empowerment, diabetes-related distress, satisfaction with treatment, barriers to medication taking, satisfaction with access to chronic care and healthcare communication, and perceived social support than men. On the other hand, women showed higher adherence than men to self-care activities dedicated to self-monitoring of blood glucose and foot monitoring. The comparison of scores between genders after adjusting for clinical and socio-demographic characteristics showed that the difference in empowerment scores was no longer significant, while all the other differences remain consistent with the crude scores (Table 3). One in four women and one in ten men showed likely depression (WHO-5 < =28), while two in three women and one in two men showed high diabetes-related distress (PAID-5) (Fig. 2).

**Table 3:** Person-centered indicators in men and women with type 2 diabetes. Crude data are expressed as mean and standard deviation (std); adjusted data are expressed as mean and standard error (se)

		Crude scores			Adjusted scores ^a^		
Person-centered care indicator	Person-centered indicator	Men	Women	p-value	Men	Women	*p*-value
		*Mean (std)*	*Mean (std)*		*Mean (se)*	*Mean (se)*	
Physical functioning	SF12 – PCS	44.9 (9.1)	40.3 (9.9)	<0.0001	44.0 (0.32)	40.8 (0.37)	<0.0001
Psychological well-being	WHO-5	62.5 (21.1)	49.5 (23.7)	<0.0001	61.2 (0.80)	50.7 (0.96)	<0.0001
Self-care activities	DSCA-Diet	5.0 (1.9)	5.0 (2.0)	0.79	4.9 (0.07)	5.0 (0.09)	0.26
	DSCA-Exe	3.4 (2.6)	2.7 (2.5)	<0.0001	3.1 (0.09)	2.7 (0.11)	0.002
	DSCA-SMBG	3.8 (2.6)	4.3 (2.5)	<0.0001	3.8 (0.09)	4.2 (0.10)	0.004
	DSCA-Feet	3.2 (2.8)	3.8 (2.8)	<0.0001	3.1 (0.10)	3.9 (0.12)	<0.0001
	DSCA-Drugs	6.6 (1.4)	6.6 (1.4)	0.89	6.6 (0.05)	6.6 (0.06)	0.88
Empowerment	DES	80.3 (15.3)	78.4 (16.2)	0.004	78.5 (0.56)	77.8 (0.66)	0.46
Diabetes distress	PAID-5	42.0 (26.9)	51.4 (28.1)	<0.0001	41.8 (1.00)	49.4 (1.19)	<.0001
Satisfaction with treatment	GSDT	80.7 (11.9)	78.8 (12.9)	0.0004	80.8 (0.44)	79.1 (0.53)	0.01
Barriers to medication taking	BM	24.4 (9.1)	26.0 (10.5)	<0.0001	24.1 (0.33)	25.7 (0.39)	0.003
Experience of access to chronic illness care	PACIC	74.8 (15.8)	73.5 (16.5)	0.05	74.3 (0.60)	72.4 (0.71)	0.05
Experience of health care communication	HCCQ	88.8 (14.2)	87.4 (15.4)	0.03	88.4 (0.52)	86.9 (0.62)	0.06
Perceived social support	PSS	81.1 (15.4)	78.2 (15.1)	<0.0001	80.8 (0.52)	77.3 (0.63)	<.0001

^a^ adjusted for age, diabetes duration, *BMI* diabetes complications, glucose-lowering treatment scheme, school education, and living status

In a multivariate analysis, factors associated with an increased likelihood of having elevated HbA1c levels (≥8.0%) were different in men and women (Table 4). In particular:

**Table 4:** Factors associated with increased likelihood of poor metabolic control (HbA1c > =8.0%)

*Covariates* ^a^	*Categories*	*Men*		*Women*	
		*OR*	*95%CI*	*OR*	*95%CI*
AGE by 5 years		**0.97**	**0.95–0.99**	0.98	0.96–1.00
School education	High school or university	**0.87**	**0.80–0.95**	0.92	0.82–1.03
	Middle school	0.95	0.87–1.03	0.94	0.84–1.05
	Primary school	1.00	-	1.00	-
Living status	Spouse / sons	1.00	-	1.00	-
	Alone	0.89	0.77–1.03	1.08	0.97–1.22
	Other	1.01	0.88–1.15	1.14	0.93–1.39
Smoker	Ex	0.99	0.92–1.07	1.05	0.94–1.17
	Yes	0.97	0.89–1.07	1.03	0.89–1.20
	No	1.00	-	1.00	-
BMI	0–27.0	**0.91**	**0.84–0.99**	0.93	0.84–1.03
	27.1–30.0	**0.89**	**0.82–0.97**	0.91	0.82–1.01
	>30.0	**1.00**	-	1.00	-
Diabetes duration	<=5.0 years	**1.31**	**1.20–1.43**	**1.33**	**1.20–1.48**
	5.1–10.0 years	**1.26**	**1.15–1.38**	**1.13**	**1.01–1.26**
	>10.0 years	1.00	-	1.00	-
Treatment class	OHAs + insulin	**1.31**	**1.20–1.43**	**1.33**	**1.20–1.48**
	Insulin only	**1.26**	**1.15–1.38**	**1.13**	**1.01–1.26**
	OHAs only	**1.00**	**-**	**1.00**	**-**
No. of diabetes complications	> = 1	0.94	0.87–1.01	0.94	0.86–1.03
	0	1.00	-	1.00	-
SF12 – PCS	Tertile 1	1.04	0.95–1.14	0.98	0.88–1.09
	Tertile 2	1.01	0.93–1.10	0.93	0.84–1.02
	Tertile 3	1.00	-	1.00	-
WHO-5	<=28	1.03	0.92–1.16	1.01	0.92–1.12
	>28	1.00	-	1.00	-
DES	Tertile 1	1.09	0.99–1.19	1.07	0.96–1.20
	Tertile 2	1.00	0.92–1.08	0.91	0.82–1.00
	Tertile 3	1.00	-	1.00	-
PAID-5 > 40	Yes	1.00	0.93–1.08	**1.15**	**1.05–1.25**
	No	1.00	-	**1.00**	**-**
GSDT	Tertile 1	1.24	0.93–1.65	0.91	0.62–1.33
	Tertile 2	1.09	0.99–1.19	1.07	0.96–1.20
	Tertile 3	1.00	0.92–1.08	0.91	0.82–1.00
PACIC	Tertile 1	1.02	0.93–1.11	0.87	0.77–1.00
	Tertile 2	0.94	0.87–1.03	0.92	0.82–1.02
	Tertile 3	1.00	-	1.00	-
HCCQ	Tertile 1	0.93	0.87–1.00	1.08	0.98–1.18
	Tertile 2–3	1.00	-	1.00	-
PSS	Tertile 1	1.02	0.93–1.11	1.02	0.91–1.13
	Tertile 2	0.99	0.92–1.07	1.00	0.90–1.12
	Tertile 3	1.00	-	1.00	-

^a^ Covariates tested in the fully adjusted model: Age, school education, living status, smoking, BMI, diabetes duration, glucose-lowering treatment class, presence of diabetes complications, PAID-5 > 40, WHO-5 < =28, and tertiles of SF12 – PCS, DES, GSDT, PACIC, HCCQ, and PSS. Tertiles were identified separately for men and women. Tertiles 2 and 3 coincided for HCCQ scores in both genders due to the extreme skewness of the distribution. Covariates related to statistically significant associations in men or women are in bold

- Both in men and in women, insulin treatment alone or in association with OHAs increased the likelihood of having HbA1c ≥ 8.0% from 13 to 33% vs. OHAs only;
- In men but not in women, the likelihood of having HbA1c ≥ 8.0% decreased as age increased (−3% for each additional 5 years) and was associated with the level of school education;
- In women but not in men, high levels of diabetes related distress were associated with a higher likelihood of poor metabolic control.

## Discussion

Data from the BENCH-D study provide new insights into the quality of care and quality of life as perceived by men and women with T2DM. Health related quality of life represents not only a key outcome of any person-centered chronic care model, but also an important mediator for adherence to treatment and the achievement of therapeutic goals.

Our data show, in line with the results of AMD-Annals [6], that women with T2DM have poorer intermediate outcome indicators than men, i.e. higher levels of HbA1c and poorer control of key cardiovascular risk factors such as BMI and LDL-cholesterol. Data also show that, compared to men, women have lower levels of general well-being, diabetes-related quality of life, satisfaction with treatment and care, self-care attitudes dedicated to physical activity, and empowerment. On the other hand, women report a higher level of adherence to SMBG and foot monitoring. It has been documented that SMBG not supported by adequate education to develop skills to modify therapy and behaviors based on the SMBG readings can worsen quality of life [33].

Our data also show that gender differences in the level of empowerment are largely dependent on the differences in the socio-demographic and clinical characteristics, while all the other person-centered indicators remain less satisfactory in women than in men also after adjustment for patient case-mix. This suggests that the lower socioeconomic status of women with T2DM in our sample was associated with autonomy and informed decision-making, as well as problem solving and goal setting (i.e. the areas covered by the DES scale) [34], but not with the general and diabetes-specific dimensions of quality of life and patient satisfaction investigated through all the other questionnaires.

Finally, at multivariate analysis adjusted for all the patient characteristics, in men the likelihood of poor glycemic control (HbA1c ≥8.0%) was associated with socio-demographic and clinical characteristics, i.e. age, school level, and insulin treatment (to be intended as a proxy of diabetes severity), while in women insulin treatment and diabetes-related distress, but not socio-demographic characteristics played a role.

Patients with higher diabetes-related distress put a greater weight on their disease, which absorbs daily mental and physical energy. Higher diabetes related emotional distress levels are associated with lower patient adherence and empowerment levels [35, 36]. Our data show that diabetes distress plays a role as a correlate of good metabolic control in women but not in men.

The study suggests that new gender-specific approaches to cope with clinical and psychosocial aspects of diabetes may be needed. The interplay among clinical and non-clinical factors is complex and gender influences the relationship between the different components.

The existence of gender-specific needs has been suggested by a relevant body of literature and healthcare systems are increasingly challenged to consider the different health issues of women and men in terms of prevention, clinical signs, therapeutic approach, prognosis, psychological and social impact [37].

From a mere clinical point of view, a gender-based approach in the management of T2DM must consider the different CVD profile of men and women. In fact, although the overall CVD risk is higher in T2DM men, the relative risk of coronary heart disease (CHD) is higher in T2DM women when compared to people without diabetes, with the loss of the typical oestrogen protection in the premenopausal state [38]. Diabetes is a powerful cardiovascular risk factor for both genders, but the relative risk of coronary heart disease deriving from having T2DM is higher in women than in men [39, 40]. Multiple factors are responsible for these differences, including gender-differences in metabolic control, cardiovascular risk, and treatments [41, 42], especially disparities in the routine management of LDL-C levels [43].

From a psychosocial point of view, many differences exist between men and women in beliefs, attitudes, fears and concerns about diabetes and its management [44]. In particular, women report significantly more depressive symptoms than men [45], female sex is a predictor of poor psychological outcomes [46], and depressive symptom severity is associated with poorer diet and medication regimen adherence [30]. Also, women more often experience diabetes related distress than men [47]. Recently, Fisher [35] clarified that diabetes distress is not a proxy for clinical depression but reflects an emotional response to a demanding health-related condition; conversely, major depressive disorder is a psychiatric disorder which is not content-related in so far that it does not describe pathology based on relevant causes, perturbations or contextual stressors. The investigation of diabetes related and unrelated stressors should represent an integral component of ongoing comprehensive care for all patients with diabetes [35].

Additional elements deriving from previous qualitative and quantitative studies are that: Women with diabetes showed less patient satisfaction and a lower health-related quality of life than men with diabetes [48]; men are more concerned about how diabetes affects their provider role [49], whereas women worry more about how self-care will hinder their familial responsibilities [50]; women tend to sacrifice their dietary regimen for their family’s food preferences [51]; men focused on practical aspects of SMBG whereas women focused on affective components of SMBG [44]; men find support from family and friends more helpful than do women [44]. Disease management programs for people with diabetes have been shown to save money and improve outcomes and it is recognized that they cannot ignore information about gender-specific differences [52].

In line with the cross-national design of the Diabetes Attitudes, Wishes and Needs second study (DAWN2™), BENCH-D study promotes benchmarking using psychometrically validated indicators to identify areas for improvement and best practices to drive changes that improve outcomes for people with diabetes [8]. Gender-differences in person-centered indicators identified in this analysis highlight the importance of considering gender as an important part of further research into person-centered diabetes care.

The study has strengths and limitations. As main strength, BENCH-D is the first study allowing a comprehensive evaluation of the association between clinical and person-centered care dimensions in the two genders, increasing the knowledge about the complex interplay among patient characteristics and perceptions. Main limitations of this analysis is its cross-sectional nature, that does not allow to establish a cause-effect relationship between clinical and person-centered indicators. However, the longitudinal phase of the BENCH-D study, focusing on informing quality of care improvements through person-centered outcomes, will allow for an assessment of the person-centered medicators of impacts of educational approaches in diabetes care on clinical and quality of life outcomes.

Finally, the study sample is representative of patients attending diabetes clinics, and results cannot be generalized to individuals who only attend their general practitioner.

## Conclusions

In the BENCH-D study we collected reliable information about a range of person-centered outcomes such as depression, diabetes-related distress, self-care activities, empowerment, satisfaction with treatment, barriers to medication taking, satisfaction with access to chronic care and healthcare communication, and perceived social support in routine clinical practice and were able to identify important gender differences in the impact of diabetes. Specifically, we found that in T2DM women show poorer clinical and person-centered outcomes indicators than men. Diabetes-related distress plays a role as a correlate of metabolic control in women but not in men. The study provides new information about the interplay between clinical and person-centered indicators in men and women which may guide further improvements in diabetes education and support programs.
